# Non-randomized controlled trial of the long-term efficacy of an Ecohealth intervention against Chagas disease in Yucatan, Mexico

**DOI:** 10.1371/journal.pntd.0006605

**Published:** 2018-07-02

**Authors:** Etienne Waleckx, Silvia Pérez-Carrillo, Samuel Chávez-Lazo, Rafael Pasos-Alquicira, María Cámara-Heredia, Jesús Acuña-Lizama, Fernando Collí-Balám, Javier Cámara-Mejía, Maria Jesús Ramírez-Sierra, Vladimir Cruz-Chan, Miguel Rosado-Vallado, Santos Vázquez-Narvaez, Rosario Najera-Vázquez, Sébastien Gourbière, Eric Dumonteil

**Affiliations:** 1 Laboratorio de Parasitología, Centro de Investigaciones Regionales “Dr. Hideyo Noguchi”, Universidad Autónoma de Yucatán, Mérida, Yucatán, Mexico; 2 Departamento de Control de Vectores, Servicios de Salud de Yucatán, Mérida, Yucatán, Mexico; 3 UMR 5096 ‘Laboratoire Génome et Développement des Plantes’, Université de Perpignan Via Domitia, Perpignan, France; 4 Department of Tropical Medicine, Vector-Borne and Infectious Disease Research Center, School of Public Health and Tropical Medicine, Tulane University, New Orleans, LA, United States of America; University of Texas at El Paso, UNITED STATES

## Abstract

Non-domiciliated intrusive triatomine vectors are responsible for a low but significant transmission of *Trypanosoma cruzi* to humans. Their control is a challenge as insecticide spraying is of limited usefulness, and alternative strategies need to be developed for a sustainable control. We performed a non-randomized controlled trial of an Ecohealth intervention based on window insect screens and community participation to reduce house infestation by *Triatoma dimidiata* in two rural villages in Yucatan, Mexico. Efficacy of the intervention was measured over a three years follow-up period and entomological indicators showed that the proportion of triatomines found inside houses was significantly reduced in houses with insect screens, which effectively kept more bugs on the outside of houses. Using a previously developed model linking entomological data to the prevalence of infection in human, we predicted that the intervention would lead to a 32% reduction in yearly incidence and in the prevalence of *T*. *cruzi* infection. The cost for the coverage of all the windows of a house was of comparable magnitude to what families currently spend on various domestic insecticide, and most screens were still in good conditions after three years. In conclusion, the Ecohealth approach proposed here is effective for the long-term and sustainable control of intrusive *T*. *dimidiata* vectors in the Yucatan peninsula, Mexico. This strategy may also be easily adapted to other intrusive triatomine species as well as other regions/countries with comparable eco-epidemiological settings, and would be an excellent component of a larger integrated program for the control of a variety of other vector-borne diseases, bringing additional benefits to the communities. Our results should encourage a further scaling-up of our implementation strategy in additional villages in the region.

## Author summary

Intrusive triatomine bugs such as *Triatoma dimidiata* in the Yucatan peninsula, Mexico, are responsible for the transmission of *Trypanosoma cruzi* to humans, which can lead to Chagas disease. The control of these bugs is a challenge as insecticide spraying is poorly effective, and alternative strategies need to be developed for a sustainable control. We tested a novel Ecohealth approach, based on window insect screens manufacture and installation through community participation to reduce the presence of bugs inside houses. The proportion of triatomines found inside houses was significantly reduced in houses with insect screens, which effectively kept more bugs on the outside of houses. We estimated that the intervention would lead to a 32% reduction in new cases of infection each year and in the prevalence of *T*. *cruzi* infection. The low cost of the intervention and the durability of screens further indicate that the proposed Ecohealth approach is effective for the long-term and sustainable control of intrusive *T*. *dimidiata* vectors in the Yucatan peninsula, Mexico. This strategy may also be easily adapted to other bug species and regions with similar characteristics and sould be expanded in additional villages in the region.

## Introduction

Chagas disease is a vector-borne parasitic disease causing major morbidity and mortality in the Americas, with at least 6 million persons currently infected. The burden of Chagas disease is estimated at 29 million disability-adjusted life years (DALYs) and it leads to health care costs of $24.73 billion [1, 2]. In Mexico, the Ministry of Health reports a few hundred cases every year [3], but estimates suggest that there may be over one million infected persons [4]. In the state of Yucatan, seroprevalence surveys have reported that 1–4% of the population is seropositive for *Trypanosoma cruzi*, the agent of Chagas disease [5–7].

Throughout the Americas, Chagas disease is principally controlled by residual insecticide spraying to reduce house infestation by triatomine vectors [8, 9]. However, the emergence of insecticide resistance [10] and the limited usefulness of insecticide spraying against intrusive vectors make such interventions clearly not sustainable for long-term vector control [9, 11, 12] and evidences the need for better and integrated vector control interventions [8]. For example, the Ecohealth approach (ecosystem approach to health) promotes interventions targeting the multiple determinants of disease transmission through transdisciplinary participatory research, integrating biological, ecological and social aspects of disease control [13–15]. Ecohealth strategies are emerging as more rational, sustainable, and cost-effective than vertically-organized and widespread empirical insecticide spraying, as they promote greater community responsibility and ownership of the intervention [16].

*Triatoma dimidiata* in the Yucatan peninsula, Mexico, is a good example of intrusive triatomines that transiently infest houses during the months of March-July [17–21], and which is difficult to control with insecticide [11, 20]. We previously identified some of the key determinants for house infestation by *T*. *dimidiata* in the region, which include house proximity to sylvatic areas, proximity to public lighting and the presence of domestic animals such as dogs and chicken, while housing quality or domestic practices have little relevance [22, 23]. Based on qualitative research, we assessed community knowledge, attitudes and practices related to triatomine vectors and Chagas disease and vector control [24, 25]. This information provided some clues to develop an Ecohealth intervention based on a community program of insect screen manufacture and installation for triatomine control in two villages [26]. Here, we further evaluated the efficacy of the intervention during three consecutive infestation seasons, based on entomological indicators.

## Materials and methods

### Study area

The current study was performed in the rural villages of Teya (21.05°N, 89.07°W), Sudzal (20.87°N, 88.98°W), and Bokoba (21.01°N, 89.07°W), located about 15–20 km apart in the central part of the Yucatan state in southern Mexico [24]. Climate in the region is warm and humid, with an average annual temperature of 26°C and 115 cm of rainfall. The villages are surrounded by a mixture of secondary bush vegetation and agricultural land. There is a total of 702, 509 and 570 houses in Teya, Sudzal (main village) and Bokoba, respectively, all of which have been georeferenced. The village of Sudzal also includes the communities of Tzalam, Chumbec and Kancabchén, which represent an additional 120 houses. The respective population is of about 2,000 inhabitants in both Teya and Bokoba, and 1,600 in Sudzal. All three villages are very similar and comparable in most relevant aspects, including triatomine infestation and *T*. *cruzi* seroprevalence in the population [7, 22–24].

### Study design, implementation process and monitoring

The study is a non-randomized controlled trial, in which vector control was implemented in two communities (Teya and Sudzal), and control households were those of both communities which did not participate in the intervention. Indeed, enrolment of the households in the project was voluntary and community-based, as described below. Consequently, allocation of mosquito screens was not randomized. For years 2 and 3 of follow-up, households of a third community (Bokoba), where no vector control was implemented, served as additional controls.

As many individuals and organizations as possible were initially approached in each community to discuss the project, allowing them to express their potential interest and contribution, and leading to the identification of stakeholders in each community as well as providing key information for a situation analysis [22, 24, 25]. The intervention was then designed through a series of open meetings with the different stakeholders. These included the communities, local governments, local health centers, social workers, carpenters, and community leaders. As previously described [26], different implementation strategies were discussed to identify the preferred process in each community (Teya and Sudzal).

Local carpenter workshops were identified by the communities and the local governments, to ensure better community dynamics and ownership of the intervention Two carpenter workshops were initially identified in Teya and one in Sudzal. However, one of the workshops in Teya withdrawed from the project shortly after initiating the intervention, due to lack of interest. Thus screens were manufactured and installed by one carpenter workshop in each village for most of the intervention. They arranged for home visits to each community member to take measurements of windows, delivery and installation of the screens. Since the large majority of households only had one bedroom, it was agreed that a single bedroom would be protected (on average with 2 windows).

Social workers from the municipal team (Teya) and from the health center (Sudzal) took responsibility to coordinate the activities related with the implementation of the interventions, following training by the research group. This included the organization of weekly community meetings at the city hall, coordinating the carpenters for the distribution of materials and supervising the installation of screens, and administrating a storage room provided by the local government, in coordination with the research group. Community meetings were held weekly in each village, with groups of 10–20 households, to provide Chagas disease awareness (including instructions for entomological monitoring), at the end of which households were offered to enroll in the intervention. Each attending family was also asked to bring another household to be enrolled at the subsequent meeting (neighbor or family member) to achieve a snowball effect and increase enrolment in the community. Thus, any construction in the community that was used as a house and sleeping quarter was eligible to participate in the intervention, and only constructions used for other purposes were considered ineligible (stores, offices, school, or abandoned houses). Households were then visited by the carpenters for measurements of window size and type. The social workers and the carpenters coordinated with the research team to ensure the provision of materials for screen manufacture, which were stored and administered under their supervision [26]. Screens were then installed by the carpenters in coordination with the households. Once implementation was initiated, the research team played a minimal role in these activities, which leadership was effectively transferred to the social workers [26]. However, weekly supervision of installed screens was performed to evaluate coverage. Maps of participating households were elaborated in QGis [27] to assess their geographic distribution. Because house location in the periphery and the proximity of public lights have been found to be risk factors for infestation [22, 23, 28], we measured the distance of houses participating in the intervention and of control house to the periphery of the village and to the nearest public light to assess any bias between these two groups.

### Entomological evaluation and surveillance

Following implementation of the intervention, its effects on house infestation by triatomines was monitored for three consecutive seasonal infestation periods to assess it efficacy and sustainability. A subset of houses was randomly selected for detailed follow-up, 95 to 122 with insect screens and 77 to 147 without insect screens, depending on the year and distributed evenly in the villages (S1 Fig). Control houses were selected from the same villages where screens had been installed, as well as from a third village (Bokoba) where no vector-control intervention had been implemented. Entomological monitoring was performed by community participation, as reported before [18], during 2 weeks for each village and each year during the infestation season. Briefly, households were instructed to collect any bugs they would detect inside or outside their own house while performing their normal daily activities. They were trained for the safe handling of triatomines to avoid infection, but no additional training was provided since inhabitants are rather knowledgeable of the bugs [24]. This method is more sensitive than timed manual searches by trained research personnel for the detection of low level infestation [18]. Nonetheless, because of potential difference in household participation between houses with screen and houses without screens, as well as changes in motivation over time, we also used mouse-baited traps [29] for unbiased monitoring of domestic infestation. A total of eight traps per house were used, consisting of four traps per bedroom window, with two traps on the inside and two traps on the outside of each screen/window (S2 Fig). Often, however, one of the outside traps and one of the inside traps were set on the outside and on the inside of the door, respectively. Traps were used only one night per house. They were set up in the evening and removed in the morning after collecting the bugs caught on the traps. We evaluated house infestation index (inside and outside infestation) expressed as the percentage of houses with bugs, and bug density index (inside and outside bug abundance/house) expressed as number of bugs/house, as well as the proportion of inside infestation and inside bug abundance/house.

We adopted a ‘Force of Infection’ (‘FoI’) approach (see [30] for a review of ‘FoI’ models of *T*. *cruzi* transmission) to predict the impact of the reduction in vector abundance on the risk of transmission in the studied villages. Such modelling allows calculating the prevalence and the incidence in human with respect to the so-called FoI, i.e. the per unit time probability for a susceptible human to become infected, that is commonly modelled as follow;

$$\lambda =1-{\left(1-p\right)}^{C}$$

where C stands for the number of potentially infectious contact (PIC) between a susceptible individual and infected vectors, and where p denotes the probability of transmission per PIC. The former can in turn be calculated using the typical relationship depicting vector ecology;

$$C=\frac{N_{v}*p_{v}*b*f_{h}}{N_{h}}$$

where *N_v_* and *N_h_* denote the abundance of vectors and humans, *p_v_* stands for the prevalence of *T*. *cruzi* infection in vectors, while b and *f_h_* are the vector biting rate and the proportion of those bites that are made on humans. Under the assumption that both the force of infection and the human death rate, *μ*, are constant, the prevalence in human is given by:

$$p_{h}=\frac{\lambda }{\lambda +\mu }$$

while the incidence was calculated as the product of the force of infection (*λ*) and the number of inhabitants.

We parameterized those equations using the same estimates as in Nouvellet et al. [31] but for the abundance of bugs that we estimated to be modified by the same proportion as that of inside infestation in houses with screens, in order to calculate the changes in incidence and prevalence *T*. *cruzi* infection due to the intervention. We also calculated the cost of the intervention, which included materials and labor for screen manufacture and installation, but not the labor from other stakeholders such as social workers or local governments.

A random subset of insect screens was also inspected every year to evaluate their integrity/wear over time (N = 182–244 depending on the year). Visual inspection allowed to classify each screen as (i) in perfect condition, (ii) with minor damage/wear (some small holes or tears but still effective), and (iii) major damage (major holes or tears, screen partially or totally removed, major loss of efficacy).

### Statistical analysis

Entomological data are expressed as infestation index and density index, or proportion, and are presented as mean ± 95% confidence intervals. Statistical comparisons between houses with insect screens and control houses were made by χ^2^ tests for the infestation index and by Z-tests (comparing the mean between two Poisson distributions) for the density index. Distance data are presented as mean ± SEM and are compared with t tests.

### Ethical statement

An informed consent was signed by the head of each participating household prior to inclusion in the study and performing any of the activities described. The project was approved by the institutional bioethics committee of the Autonomous University of Yucatan and of the TDR/World Health Organization.

## Results

### Implementation of the intervention

**Fig 1:**
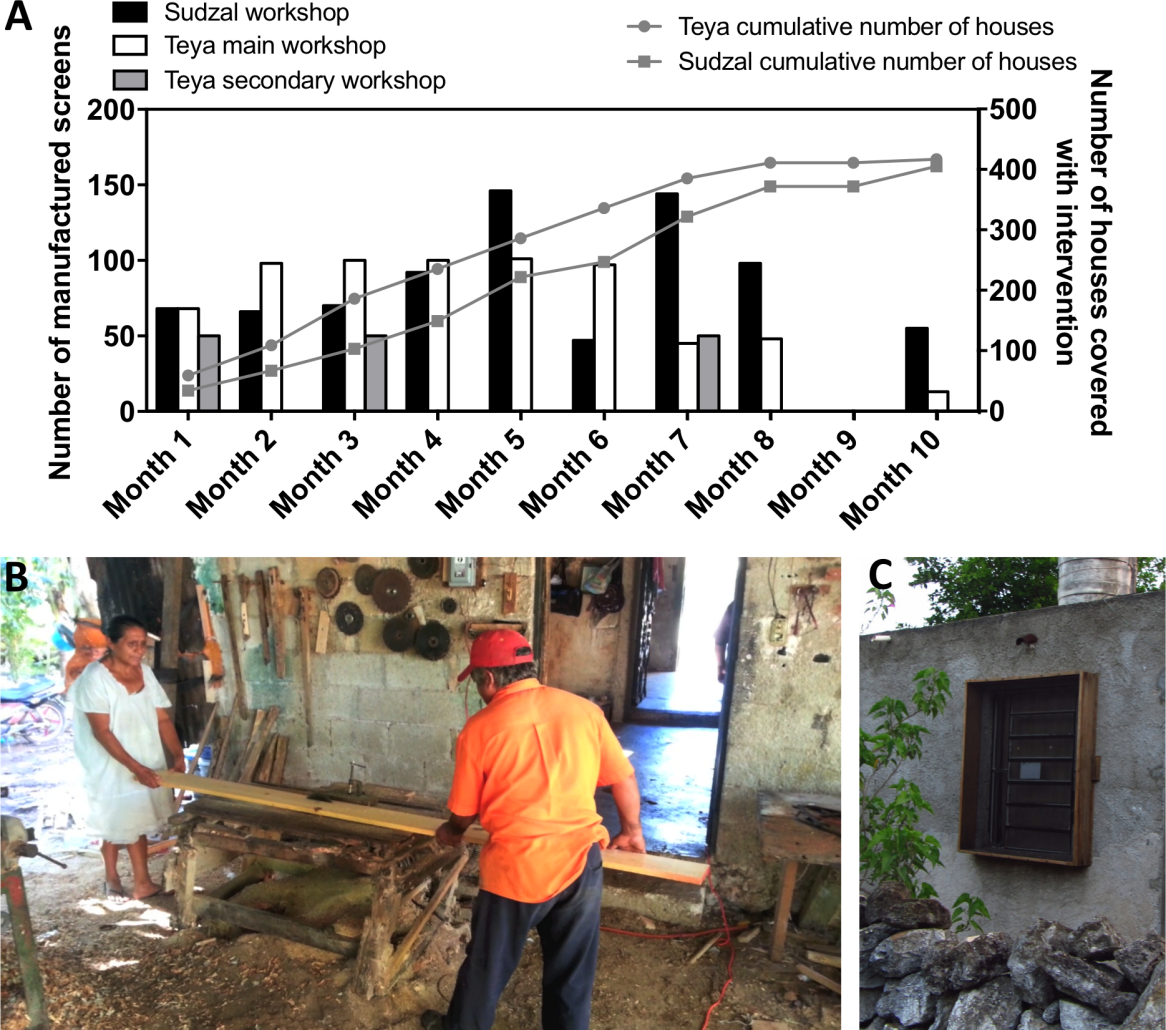
Insect screen manufacture by carpenter workshops. (**A**) Rate of screen manufacture. Monthly rate of screen manufacture and installation (left axis) is shown for the 3 participating carpenter workshops. The cumulative number of households covered by the intervention (right axis) is shown for the villages of Teya and Sudzal, respectively. (**B**) Example of carpenter workshop in Sudzal. (**C**) Example of screen design once installed.

A total of 1,606 window screens were installed in 822 households in the villages of Teya and Sudzal, over a period of about 10 months (Fig 1A). Logistical difficulties to ensure the delivery of materials to the carpenters at the beginning of the intervention caused some minor delays, as well as some difficulties in coordinating the carpenter workshop with the households for screen installation at the end of the intervention. Nonetheless, all workshops generally produced about 50–100 screens/month (Fig 1A). This included an initial visit to each participating household for measurement of window size and type, manufacture and installation of the screens. Maximum manufacture and installation rates even reached up to 150 screens/months in two occasions in Sudzal. This rate of implementation seems very good given the rudimentary facilities and limited personnel (family members) available in each workshop (Fig 1B) and the various styles and designs of the screens so that they appropriately fitted each window (Fig 1C).

The 822 households participating in the study corresponded to a coverage of the intervention of 59% of households in Teya (417/702) and 58% in Sudzal main village (295/509). However, because some households already had insect screens, and other were ineligible for the study because they were not used for sleeping (stores, offices, or abandoned), a higher proportion of houses in each village actually had screens. For example this proportion reached 76% of houses in Sudzal main village (336/440 eligible houses).

**Fig 2:**
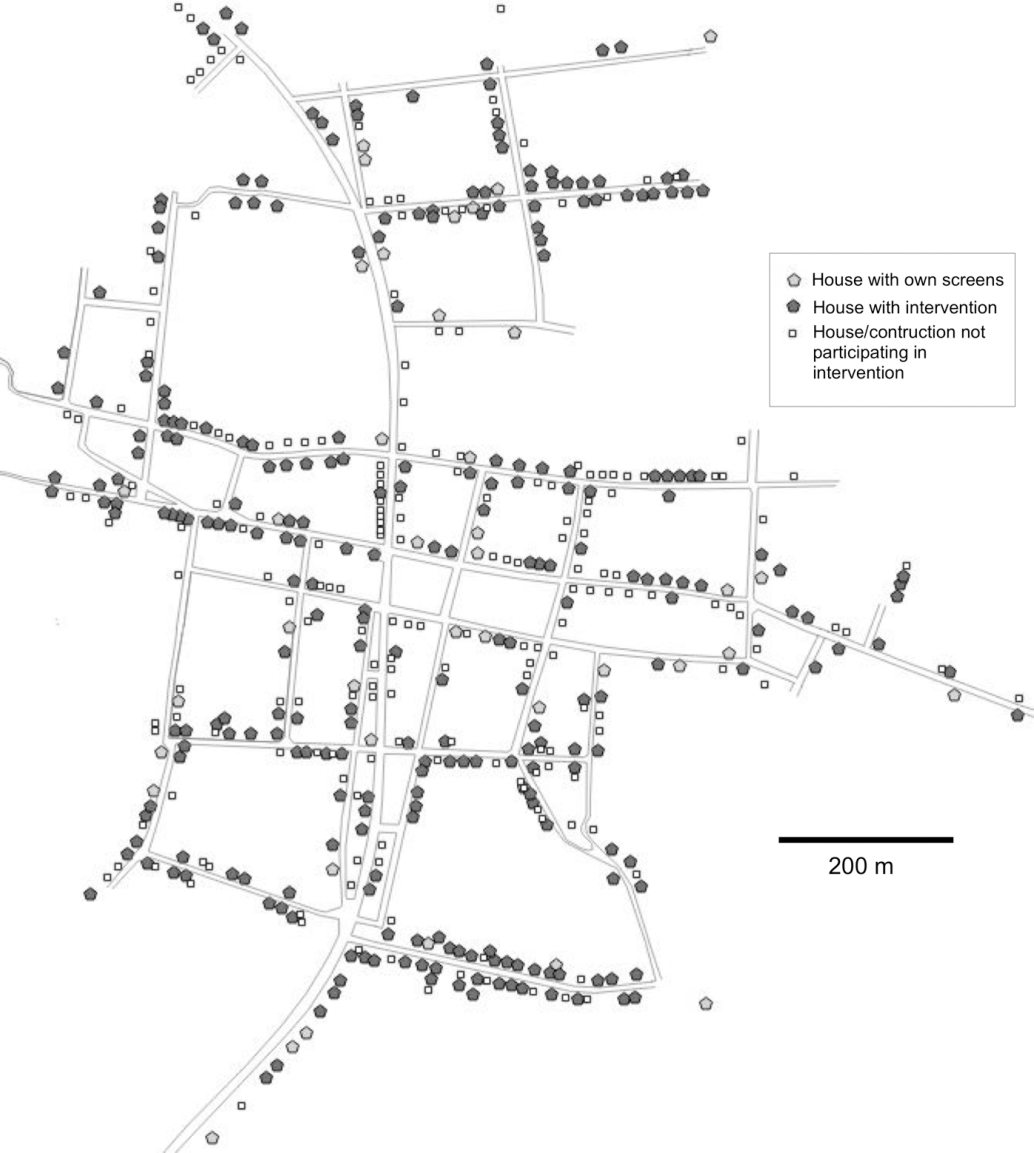
Geographic distribution of houses with and without screen intervention in Sudzal.

Participating households were distributed across the entire village in both Teya and Sudzal (Fig 2), and thus were exposed to a comparable risk of infestation as non-participating households without insect screens, due to their location within the village [23, 28]. Indeed, houses with screen intervention were located at a distance of 119 ± 10 m of the periphery of the village, while control houses not participating in the intervention were located at 114 ± 9 m of the periphery (t test, P = 0.52). Similarly, houses from the intervention were located at a distance of 24.6 ± 2.1 m from public lights, while control houses not participating in the intervention were located at 23.7 ± 3.6 m from public lights (t test, P = 0.58).

### Efficacy of the vector control intervention

Entomological surveillance was used to evaluate the efficacy of the intervention to reduce triatomine presence for three years following the installation of screens. Bugs were collected by community participation during 2 weeks per village and year of follow-up in both houses with screens and control houses that did not participate in the intervention. Houses from the village of Bokoba, which did not receive control intervention, were used as additional controls. Furthermore, mouse-baited traps were also used to complement community bug surveillance and detect potential bias in community participation. A total of 5744 traps were set inside and outside 718 houses (one trapping night/house) evaluated during the three-year follow-up (see Materials and Methods).

A total of 440 bugs were collected over the three years of follow-up. Most bugs were collected by community participation (368 bugs) compared to mouse-baited traps (72 bugs). Ninety six percent were adults (63% females and 37% males), and only 4% were nymphal instars as observed before [17, 19]. A total of 147 bugs were collected in houses without screens (both outside, i.e. front or backyard, or outside walls of the houses and inside the houses), while 220 were collected in houses with insect screens (both outside and inside). The overall infestation index (*i*.*e*. taking into account bugs collected inside and outside houses) was 18.6% in 2013 (34/183), 22.5% in 2014 (59/262), and 30.4% in 2015 (83/273), which likely reflected our multiple efforts to motivate and increase community participation to find bugs during the study. There were no significant differences in infestation among control houses from the villages of Teya, Sudzal and Bokoba *(*P = 0.538), which data were thus pooled for every year of follow-up.

**Fig 3:**
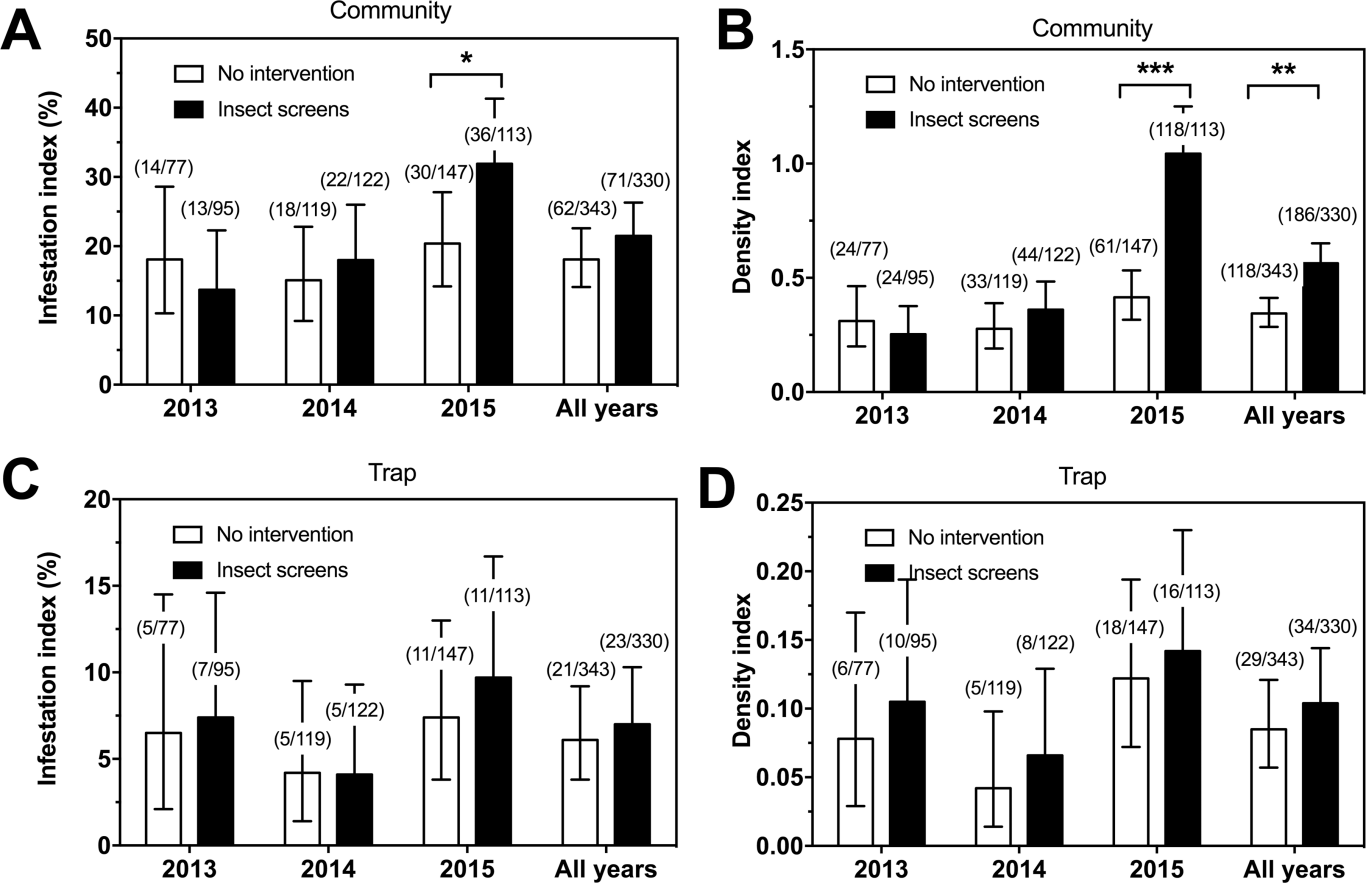
Effect of insect screens on house infestation and density of triatomines. Triatomines were collected by community participation (A and B), and by mouse-baited traps (C and D) in houses with insect screens (black bars) and control houses with no vector control intervention (white bars), during three years of follow-up of the intervention. Houses with insect screens are from the villages of Teya and Sudzal, while control houses are from the village of Bokoba, Sudzal and Teya. Infestation index (A and C) and density index (B and D) were measured and expressed as % of infested houses, and bug number/house, respectively. Data are presented as mean ± 95% confidence interval. *, ** and *** indicate significant differences between groups (P<0.05, P<0.01 and P<0.001, respectively).

Based on bug collections by community participation only, overall infestation index (inside + outside) indicated that around 15.3–26.4% of houses were infested over the three years study period, with no significant differences in infestation between houses with and without screens, except in year 3 which showed a significant increase in infestation in houses with insect screens (Fig 3A). Triatomine density index (inside + outside bugs) showed a small but significant increase from 0.34 to 0.56 bug/house for houses without screen and houses with insect screens, respectively, for the 3 years of follow-up (Fig 3B). This increase was due to a large increase in the density of bugs collected from houses with screens in year 3 of the intervention.

On the other hand, data from mouse-baited traps evidenced no significant difference in infestation index (inside + outside bugs, Fig 3C) and density index (inside + outside, Fig 3D) between houses without screens and houses with insect screens for any of the years of follow-up. As this method provides an unbiased evaluation of infestation, the increase in infestation observed by community participation in year 3 is very likely to be due to a much greater awareness of Chagas disease and motivation to participate of households who benefited from the insect screens. Indeed, we did not expect the presence of screens to reduce overall triatomine populations since these screens were not impregnated with insecticide and thus ineffective at killing bugs.

We then evaluated in more details if infestation occurred inside or outside houses, to evaluate if insect screens helped keeping bugs outside of houses. Based on community collections, both inside infestation and density indexes were constant over time with negligible yearly variations in control houses (S3A and S3B Fig). On the other hand, in houses with screens, both inside infestation and density index were lower than in control houses for years 1 and 2, but higher in year 3. Based on traps (S3C and S3D Fig), the effect of screens on inside infestation and density indexes could not be detected in years 1 and 2, but both indexes tended to be lower in houses with screens in year 3, strongly suggesting that the increase shown by community participation for that year is most likely due to and increased community participation of this group due to greater Chagas disease awareness.

**Fig 4:**
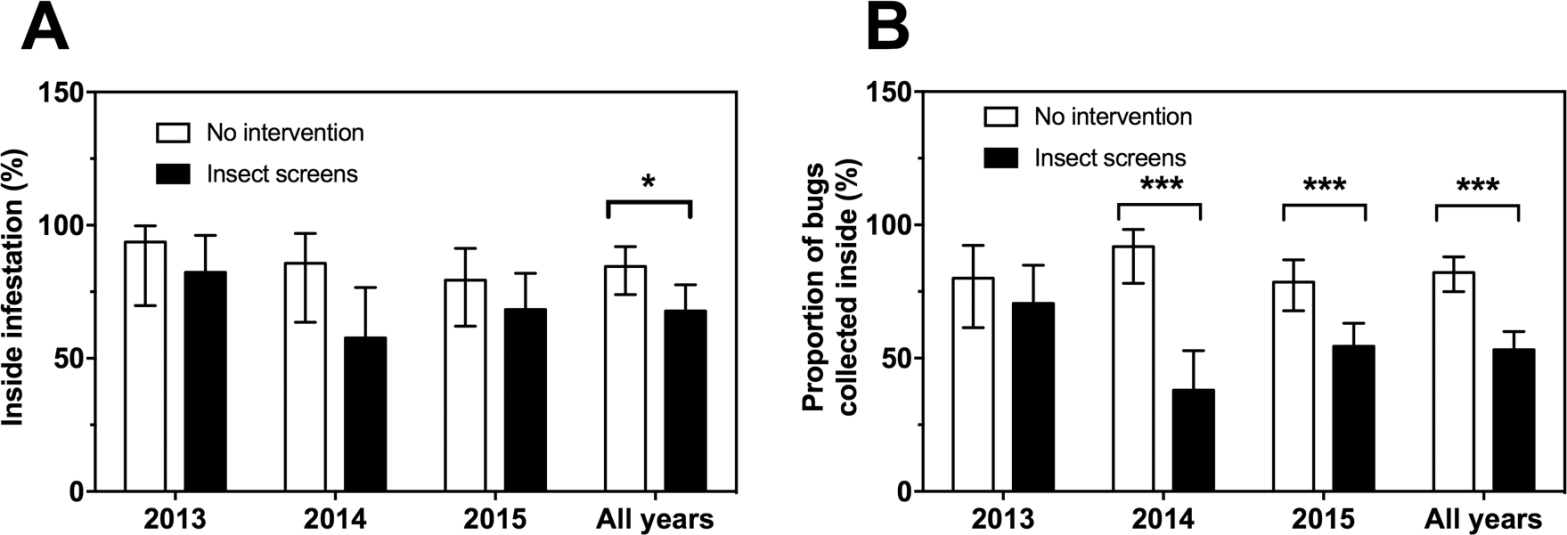
Effect of insect screens on inside infestation and density of triatomines. Triatomines were collected by community participation and by mice-baited traps in houses with insect screens (black bars) and control houses with no vector control intervention (white bars), during three years of follow-up of the intervention. Inside infestation index (**A**) and proportion of bugs collected inside (**B**) were measured. Data are presented as mean ± 95% confidence interval. * and *** indicate significant differences between groups (P<0.05 and P<0.001, respectively).

Therefore, we also assessed the efficacy of the intervention by looking at the proportion of bugs inside/outside, to control for potential bias in surveillance effort among households. Based on both community participation and mouse-baited trap bug collections, there was a small but statistically significant decrease in the proportion of houses with indoor infestation when screens were present, and a constant trend was observed each year of the follow-up period (Fig 4A). More importantly, the proportion of bugs inside houses was reduced in houses with insect screens, and this effect was statistically significant for two of the three years of follow-up (Fig 4B), as well as for the cumulative three years. Thus, the presence of insect screens effectively kept more bugs on the outside of houses.

To further assess the impact of this intervention on Chagas disease in these communities, we used a previously developed model linking entomological data to the prevalence of *Trypanosoma cruzi* infection in humans [31]. With this model, the interventions proposed in this study predicted to lead to a 32% reduction in yearly incidence and in the prevalence of *T*. *cruzi* infection. Based on a current seroprevalance of 1–4% in these villages [7], we could thus expect to reduce it to 0.7–2.7% with this vector control intervention.

**Fig 5:**
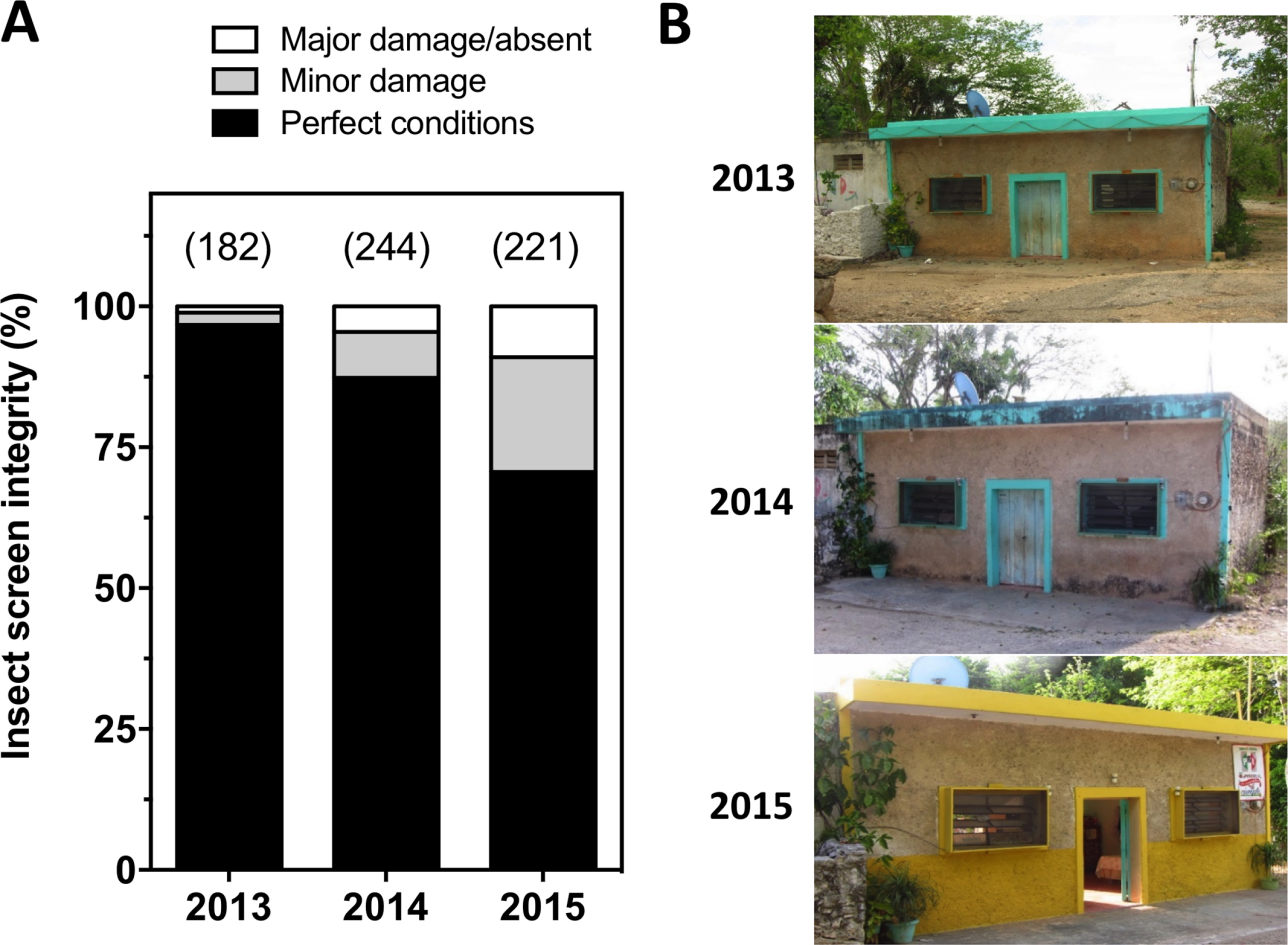
Insect screen integrity over time. Insect screens were evaluated every year for physical integrity and potential damages. (**A**) global integrity of screens, which were scored as in perfect conditions (black), with minor damage/wear (gray), and with major damage or absent (white). The number of screens evaluated each year is shown in parentheses. (**B**) Example of a house over three years.

In terms of costs, the average cost per insect screen was of 17 $US, *i*.*e*. an average cost of 34 $US per household for the coverage of one bedroom with 2 windows. About half of this cost corresponded to labor, and the other half for materials, most of which being for the wood needed for the frames of the screens (70% of material costs). We also evaluated the integrity of the screens over time, as a preliminary assessment of the long-term sustainability of the intervention. After three infestation seasons, only about 10% of the screens presented major damage to render them ineffective, and the large majority remained in perfect conditions (Fig 5).

## Discussion

Vector control against Chagas disease remains key to prevent new cases of *T*. *cruzi* infection, but has proven challenging for triatomine species behaving as intrusive bugs which transiently invade houses [9]. Previous modeling and pilot field work showed that insect screens acting as a physical barrier preventing bugs from entering houses could be a valuable control strategy in this situation [11, 12], which raised questions for the scaling-up and implementation of this kind of intervention.

We investigated here the implementation and efficacy of a village-scale intervention involving the manufacture and installation of insect screens covering two rural communities in the Yucatan, Mexico, under a community participation framework. We were able to reach a high participation and coverage in the two study villages, reaching over 70% of eligible houses within 10 months. Such a level of coverage can be considered excellent for a community-based intervention as it reached comparable levels as a systematic vertical intervention [32–34]. Engaging the community and all stakeholders in Chagas disease awareness was likely a fundamental activity allowing to reach such a high participation and coverage of the intervention. The high acceptance and desirability of insect screens as perceived by households also likely contributed [24].

In term of efficacy of the intervention to control triatomine infestation, we observed that it had no significant effect on overall (inside and outside) house infestation or bug density index, as can be expected from an intervention based on a physical barrier, which does not kill any bugs. There was even an increase in infestation and bug density in the third year of follow-up in houses with screens based on bug collections by community participation. The most likely explanation for his observation is that there was a much greater interest and thus more active entomological surveillance from households with screens compared to households who did not participate/accept the screens. This explanation is further supported by bug collection data with mouse-baited traps, which provide an unbiased assessment of triatomine infestation, and confirmed that there were no differences of the total density of bugs found inside and outside between houses with and without insect screens. However, when we analyzed infestation in further detail and assessed if bugs were found inside or outside houses, it became clear that insect screens effectively worked as physical barriers limiting bug entry inside houses and maintaining them outside. Importantly, with only two windows covered by screens, we observed a significant reduction in bug abundance inside houses, and we estimated that the concomitant reduction in vector-host contacts would translate into a 32% reduction in yearly incidence and in the prevalence of *T*. *cruzi* infection in humans. Such a reduction is highly significant considering that doors (which often remain open at this very hot time of the year), and in some cases additional windows, were left unprotected. We can thus expect that a more complete coverage of houses with screens on all openings would allow reaching an even greater efficacy. Indeed, our previous pilot testing of insect screen covering all windows allowed tor reach 87–100% efficacy in reduction in triatomine collections over a 2 years follow-up [12].

In terms of cost, we reached an average of 17 US$ per screen, although this varied according to size and type of screen, corresponding to about 34 US$ per household. This is somewhat lower than the cost of screens used in our previous pilot study [12], and can be attributed to the bulk prices for materials that we were able to obtain for large-scale purchases. Since most houses have a total of 3–4 windows when considering all rooms [22], the cost for a complete coverage of all the windows would reach 51–68 US$. Importantly, this is of a comparable magnitude to what families currently spend on various domestic insecticide such as plug-in repellent, repellent coils, or insecticide sprays, which amount to an average of 32 $US/year [24]. This suggest that while one of the main barrier for families to purchase their own insect screen is their perceived excessive cost, specific education may help them reconsider and redirect their spending from insecticide products to insect screens [24].

The long durability of the screens observed in our study further indicate that the proposed intervention would be highly sustainable, allowing to maintain a barrier effect for many years with minimal screen maintenance. Indeed, while no specific instructions were provided to households for the care and maintenance of their screens, the large majority remained in perfect condition after three years. Screens would likely need to be replaced only every 5–10 years, making the intervention very cost-effective. In addition such insect screens would not only allow for the control of triatomine bugs, but would also be effective against mosquitoes, thus preventing Dengue, Chickungunya, or Zika virus infections [35], which are also major public health issues in the region. Therefore, this intervention could be part of an integrated program for vector-borne disease control, further increasing its cost-effectiveness.

Our study presents nonetheless some limitations. First the intervention allocation was not randomized, as our objective was to cover the entire community in each village, as this provided some estimates of potential coverage of the community-based enrollment for further scaling up of the proposed intervention. Also, only bedrooms were considered for the installation of screens, due to limited resources, which may have lead to some underestimation of screen efficacy. Finally, the increased bug collections in intervened houses that we interpreted as an increase in awareness of these households generated some bias for the evaluation of entomological efficacy of the insect screens, that needed to be controlled for. On the other hand, this also provided evidence of the successful mobilization of the community for more active vector surveillance.

In conclusion, the Ecohealth approach that we developed here and the proposed community intervention based on the installation of insect screens appears to be effective for the long-term and sustainable control of invasive *T*. *dimidiata* vectors in the Yucatan peninsula, Mexico. This approach and strategy may also be easily adapted to other invasive triatomine species as well as other regions/countries with comparable eco-epidemiological settings, and would be an excellent component of a larger integrated program for the control of a variety of other vector-borne diseases, bringing additional benefits to the communities. Therefore, our results should encourage a further scaling-up of our implementation strategy in additional communities in the region. The additional targeting of additional risk factors for triatomine infestation, such as public lights or the presence of domestic animals may be explored to further increase the efficacy of the intervention.

## Supporting information

S1 Fig Map of houses participating in entomological surveillance during the three years follow-up in the village of Sudzal. (TIF)

S2 Fig Mouse-baited traps used for unbiased monitoring of inside/outside infestation of houses. (A) Example of the location of the 4 traps per screen/window. (B) Positive outside trap with an adult specimen of *T*. *dimidiata*.
(TIF)

S3 Fig Effect of insect screens on house inside infestation and inside density of triatomines. Triatomines were collected by community participation (A and B), and by mouse-baited traps (C and D) in houses with insect screens (black bars) and control houses with no vector control intervention (white bars), during three years of follow-up of the intervention. Houses with insect screens are from the villages of Teya and Sudzal, while control houses are from the village of Bokoba, Sudzal and Teya. Infestation index (A and C) and density index (B and D) were measured and expressed as % of infested houses, and bug number/house, respectively, taking into account only bugs collected inside houses. Data are presented as mean ± 95% confidence interval.
(TIFF)
