# Supplementary figures and images for: Non-randomized controlled trial of the long-term efficacy of an Ecohealth intervention against Chagas disease in Yucatan, Mexico
Source: PLoS Negl Trop Dis. 2018 Jul 2;12(7):e0006605. doi: 10.1371/journal.pntd.0006605 (PMC6044551; doi:10.1371/journal.pntd.0006605)

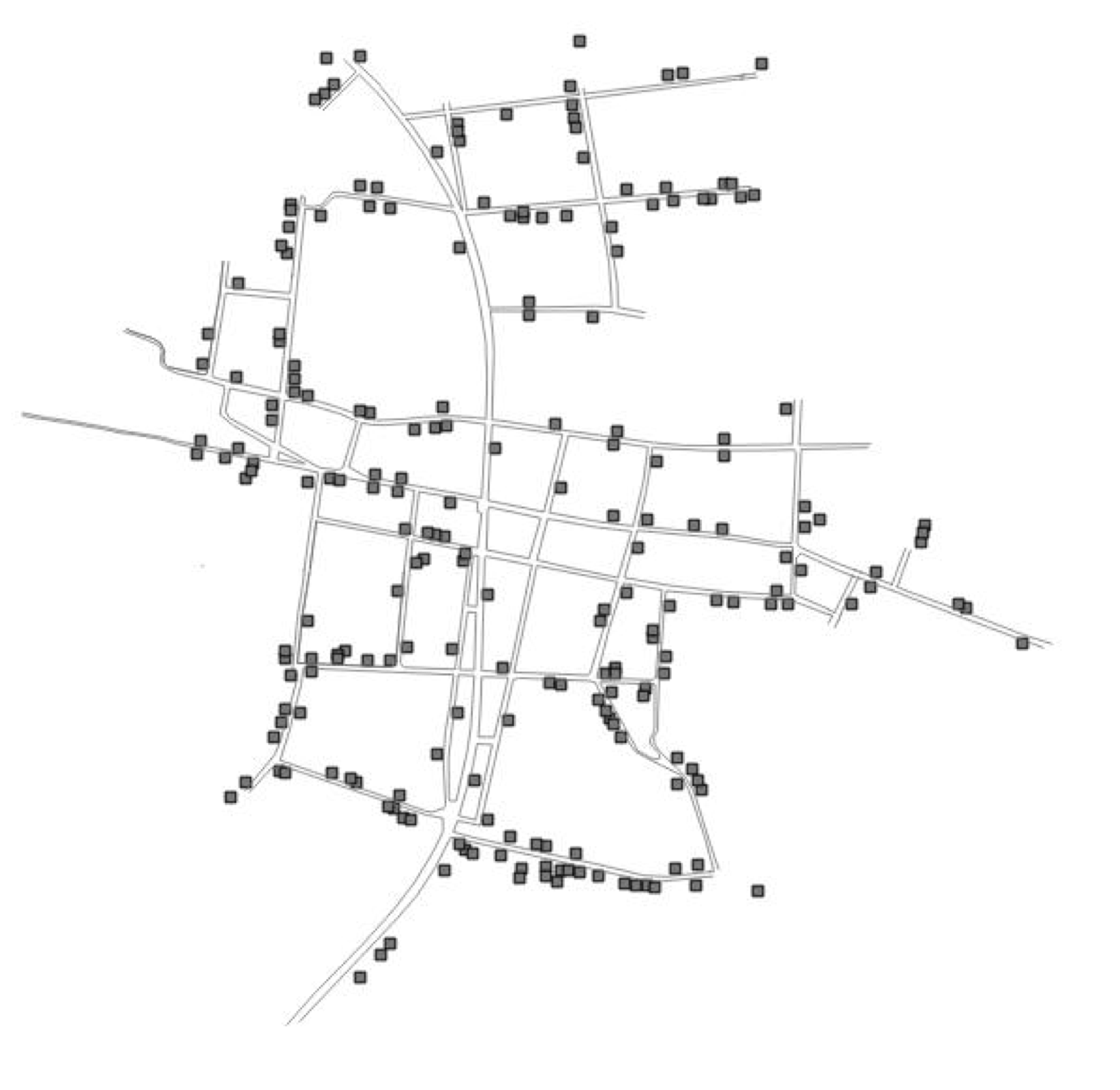

Supplement: S1 Fig — (TIF) [file pntd.0006605.s001.tif]

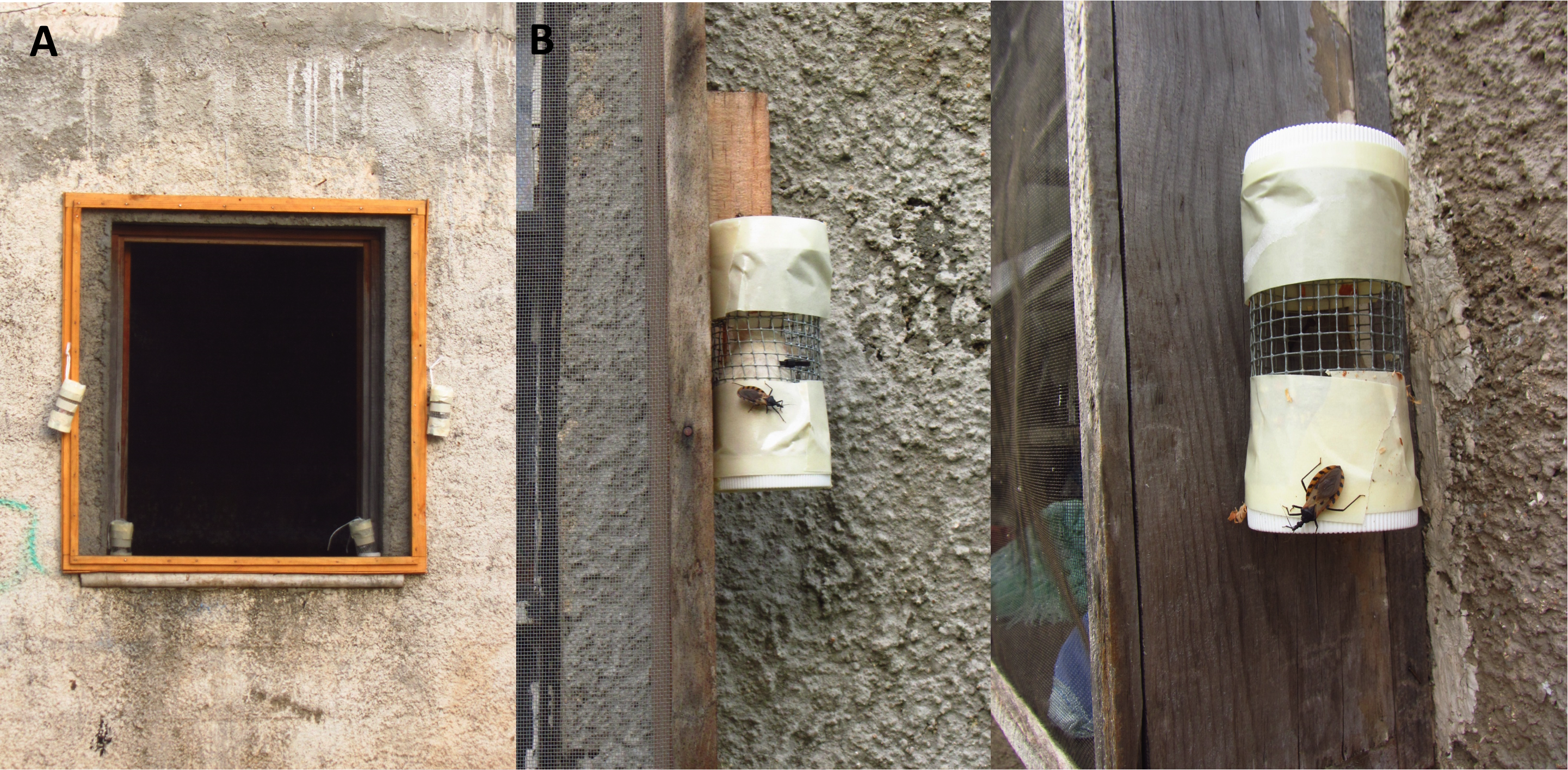

Supplement: S2 Fig — (A) Example of the location of the 4 traps per screen/window. (B) Positive outside trap with an adult specimen of T. dimidiata. (TIF) [file pntd.0006605.s002.tif]

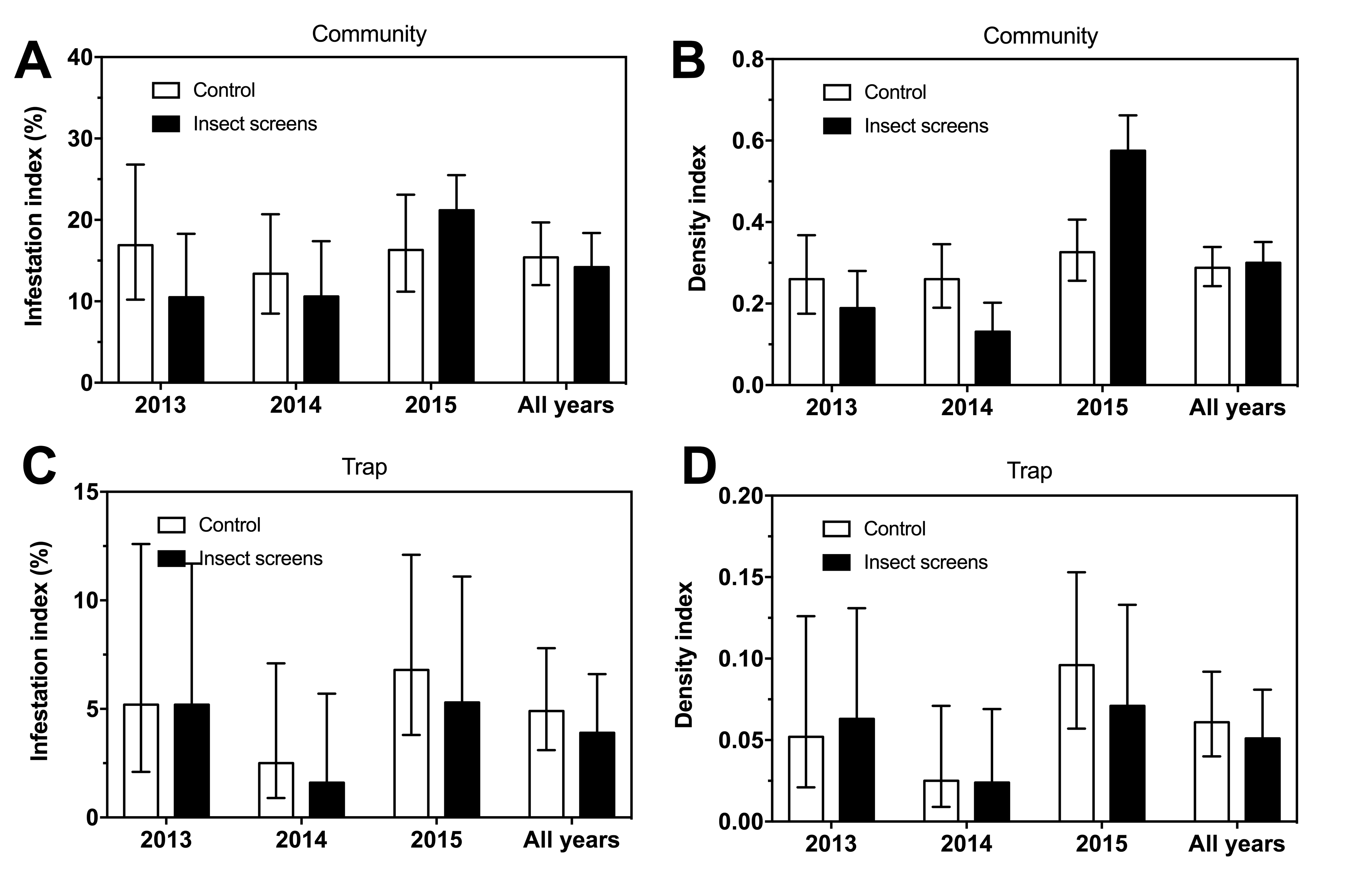

Supplement: S3 Fig — Triatomines were collected by community participation (A and B), and by mouse-baited traps (C and D) in houses with insect screens (black bars) and control houses with no vector control intervention (white bars), during three years of follow-up of the intervention. Houses with insect screens are from the villages of Teya and Sudzal, while control houses are from the village of Bokoba, Sudzal and Teya. Infestation index (A and C) and density index (B and D) were measured and expressed as % of infested houses, and bug number/house, respectively, taking into account only bugs collected inside houses. Data are presented as mean ± 95% confidence interval. (TIFF) [file pntd.0006605.s003.tiff]
